# Cavitand-Mediated Photodimerization of Chalcones: The Effect of Supramolecular Influences and Temperature on Reaction Selectivity

**DOI:** 10.3390/molecules31060983

**Published:** 2026-03-15

**Authors:** Joydip Chatterjee, Mahesh Pattabiraman, Debajit Chakraborty, Aleksander L. Wysocki, Frank Kovacs

**Affiliations:** 1 Department of Chemistry, University of Nebraska at Kearney, Kearney, NE 68849, USA; joydipchatterjee2017@gmail.com (J.C.); kovacsfa@unk.edu (F.K.); 2 Department of Physics and Astronomy, University of Nebraska at Kearney, Kearney, NE 68849, USA; chakrabortyd@unk.edu (D.C.); wysockia@unk.edu (A.L.W.)

**Keywords:** photochemistry, supramolecular chemistry, chalcones, host-guest, cyclodextrin, cucurbiturils, cyclobutane, photocycloaddition

## Abstract

The photocycloaddition (PCA) of chalcones represents an important reaction pathway for accessing substituted cyclobutanes, which is a molecular framework with utility in synthetic chemistry, materials science, and medicine. In the past, our group has demonstrated the utility of the large cavity of γ-CD as a container for encapsulating two photo reactants for directing the PCA of several classes of aryl alkenes with high stereo- and regioselectivity: the cavitand-mediated photodimerization (CMP) approach. The CMP of chalcones reported in this work further demonstrates the effectiveness of this approach as high yields of dimers were observed in the photoreactions, while they were non-reactive in the solid state and yielded only the isomerization product in homogeneous media. The γ-CD CMP of chalcones yielded predominantly dimerized products in very good to high yields (>70%), composed of a mixture of three dimers in different proportions with *syn* HH as the major product. Computational analysis of the ground state complex structures revealed a strong correlation between the stability of the complex and predominance of the stereoisomer in the mixture. Further insights were deduced from temperature-dependence studies, which showed a shift in dimer selectivity tending towards a single stereoisomer.

## 1. Background

Photodimerization reactions offer a pathway to synthesize complex molecular architectures from alkenes, leveraging light as a renewable energy source [1,2,3]. These reactions are not only fundamental to organic photochemistry but also have implications for organic synthesis [1,4], developing novel materials [5,6], sensors [7] and polymers [6,8,9], molecular biology [10,11], and medicine [12]. However, photodimerization is typically the less-preferred reaction pathway for alkenes, as its entropically unfavorable bimolecularity is precluded by unimolecular photoisomerization chemistry. Several approaches have been developed in the past to control the excited state reactivity of alkenes to produce dimers, and these methods have been further honed to affect the stereo- and regioselectivity of dimers. We, among others, have utilized the confined spaces within macrocyclic cavitands (host–guest approach) to encapsulate and orient photoactive alkenes to affect high-yield photodimerization reactions and achieve high dimer stereo- and/or regioselectivity [13,14,15,16,17].

**Figure 1:**
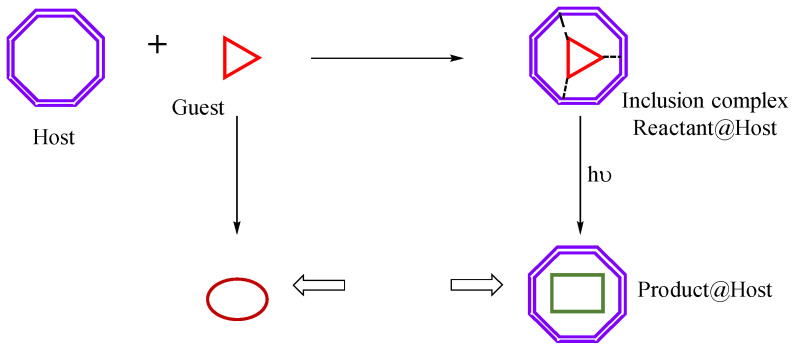
Representation of host–guest inclusion complex formation, and supramolecular interactions leading to differential reactivity, compared to free guest.

The host–guest approach to directing and controlling photodimerization has been utilized to affect the reaction in a wide range of bimolecular reactions. We have pursued this chemistry in a systematic manner with the aim of understanding the various supramolecular and photochemical factors that control reaction outcomes. Our group has delved into the photodimerization processes of reactants such as stilbenes, cinnamic acids, coumarins, aryl acrylic acids, and dibenzal acetones, within the confines of cyclodextrins and cucurbiturils [14,18,19,20]. These studies have consistently revealed that γ-*CD* and cucurbit[8]uril (CB8) can influence the selectivity and efficiency of photodimerization reactions, yielding predominantly a single product under optimized conditions. This selectivity is attributed to the ability of these cavitands to encapsulate two guest molecules simultaneously in a manner that predisposes them to specific reaction pathways (Figure 1).

**Figure 2:**
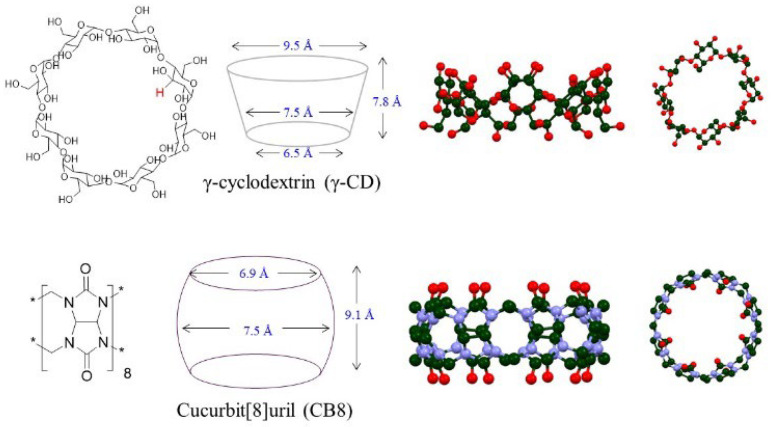
Chemical structures, simplified line-drawing, and molecular modeling representations of macrocyclic hosts used in this study. Atoms are color-coded for elements: red atoms are oxygen, blue atoms are nitrogen, and dark green atoms are carbon.

Despite the established utility of large macrocyclic cavitands in mediating photodimerization reactions, the behavior of chalcones within these supramolecular systems has not been extensively explored. Preliminary observations suggest that chalcones, when subjected to photodimerization within cyclodextrin cavities, exhibit a distinct selectivity that deviates from the patterns observed with other substrates. This deviation implies a novel interplay of supramolecular forces at work, potentially arising from the unique structural features of chalcones and their interaction dynamics with cyclodextrins (Figure 2).

Supramolecular photocyclodimerization within γ-cyclodextrin cavities has been extensively investigated using 2-anthracenecarboxylic acid as a model system. Early studies demonstrated that γ-cyclodextrin forms a 2:1 inclusion complex that enables control over head-to-tail versus head-to-head selectivity as well as enantiodifferentiation of the resulting cyclodimers [21]. Subsequent work showed that chemical modification of the host cavity can invert product distribution and enhance stereocontrol through host modification and steric effects [22]. Later reports involving systematic investigations demonstrated that supramolecular photocyclodimerization can be highly sensitive to external steric perturbations, host identity, temperature, and pressure, which collectively influence the geometry and population of precursor inclusion complexes within confined environments [23,24]. Collectively, these studies highlight the sensitivity of confined photochemical reactivity to subtle thermodynamic and environmental perturbations.

The present study aims to elucidate the mechanisms underlying the photodimerization of chalcones within γ-CD environments, focusing on the influence of these supramolecular systems on reaction selectivity and the stereochemical outcomes of the dimerization process. Through a comprehensive series of experiments and detailed analytical and computational investigations, we seek to unravel the intricate balance of forces within γ-CD complexes that govern the observed selectivity. By shedding light on (pun intended) these supramolecular interactions, this research not only contributes to the fundamental understanding of host–guest chemistry but also highlights the potential of cyclodextrins as powerful tools in the precise control of chemical reactions.

## 2. Excited State Chemistry of Chalcones

**Figure 3:**
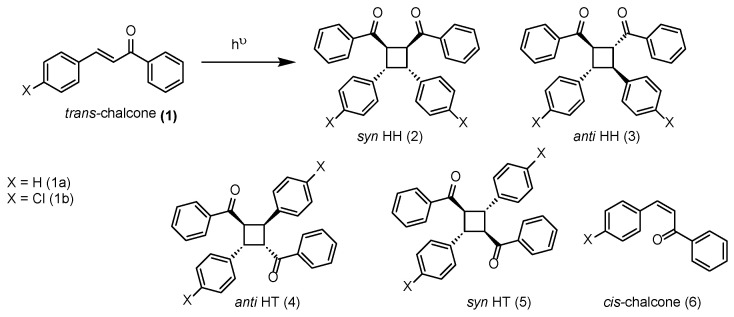
Photochemistry of chalcone leading to dimerized and isomerized product(s).

The excited-state chemistry of chalcone (Ph–CO–CH=CH–Ph) and its para-substituted analogs is understood to predominantly involve π–π* transitions upon UV or visible-light excitation [25]. In the absence of confinement or sensitization, these systems are prone to rapid *cis–trans* isomerization, which serves as a competitive relaxation pathway and significantly diminishes the efficiency of overall photochemical yield. Toda and co-workers demonstrated that direct irradiation of chalcones in solution results in poor yields of [2+2] dimers [26], with the *anti* HH product forming only in trace amounts, and the inefficiency attributed to the fast geometric isomerization of the excited chalcone effectively outpacing bimolecular PCA in unrestricted solution media. Lei et al. further substantiated this limitation by showing that under direct UV light, the photodimerization of chalcone proceeds with only ~6% yield [27]. However, by employing visible-light triplet sensitization using Ir(ppy)_3_ (*E_T_* ≈ 58 kcal/mol), they achieved selective and high-yielding formation of the anti-head-to-head dimer via energy transfer to the triplet state of chalcone. Time-resolved phosphorescence experiments revealed a long-lived chalcone triplet emission at 590 nm (τ ≈ 80 µs), which facilitates a diffusion-limited intermolecular reaction with ground-state chalcone molecules. DFT calculations supported a mechanism involving *trans*–*trans* preorganization, diradical intermediate formation, and subsequent ground-state cyclization, explaining the observed regio- and diastereoselectivity (Figure 3).

Taken together, these studies emphasize that successful [2+2] photodimerization of chalcones requires either electronic modification of the excitation pathway (such as triplet sensitization) or physical control over molecular orientation and proximity. In both cases, minimizing the influence of isomerization and maximizing reactive alignment is essential. This reinforces the conceptual basis for supramolecular strategies such as host–guest confinement using CDs to maneuver chalcone reactivity towards selective dimerization over competitive reaction and/or deactivation pathways.

The supramolecular photochemistry of chalcones remains relatively underexplored compared to that of cinnamic acids and stilbenes, particularly for minimally substituted chalcones. Herein, we report the inclusion complex formation and photochemical reactivity of chalcones within γ-cyclodextrin, including detailed analysis of product distribution, stereochemical selectivity, and spectral characteristics of the isomeric dimers formed. Additionally, we present the effect of temperature on product selectivity which has provided interesting and deep insights into the dynamic supramolecular influences affecting reaction dynamics.

## 3. Results and Discussion

***Inclusion Complex Titration:*** was performed as a first step to understand the complexation dynamics of the guests to γ-CD and followed with ^1^H NMR spectroscopy. Chalcone **1a** is sparingly soluble in water and sonication of the compound in D_2_O in a heated sonic bath for two hours resulted in a saturated solution of sufficient concentration to yield observable proton signals in ^1^H NMR spectroscopy (Supplementary Materials, Section S2). Addition of 0.5 equivalent of γ-CD to the saturated chalcone solution resulted in a significant upfield shift in the chemical shifts and peak broadening of the proton signals of the guest indicating inclusion of the guests within the host cavity. In addition, a noticeable increase in the signal intensity of the guests was also observed, which suggested an enhancement of solubility mediated by the host in the aqueous medium.

**Figure 4:**
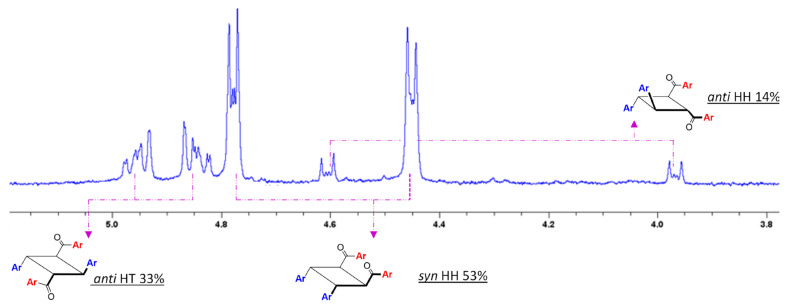
Partial ^1^H NMR of reaction mixture from irradiation of ternary complex of chalcone **1a** encapsulated within γ-CD (1a_2_@ γ-CD). The displayed region corresponds to the cyclobutane proton signals. Full spectrum is provided in SI Figure S4. Solvent is CDCl_3_.

***CMP of Chalcones:*** The chalcones used in the study formed a stable inclusion complex with γ-CD when stirred in aqueous medium as evidenced by the off-white slurry that results from the mixture of the individual components as outlined in Section 4. Irradiation of the slurry for 24 h resulted in complete loss of color as the slurry turned completely white, indicating loss of extended conjugation in the chromophore, decreasing its absorption in the visual spectrum. The reactants and the products were extracted from the host–guest complex with ethyl acetate, the solvent removed under vacuum, and the components analyzed spectroscopically. The ^1^H NMR spectrum of the reaction mixture acquired in CDCl_3_ showed the presence of three sets of cyclobutane signals (Figure 4) of the dimeric products appearing distinctly in the midfield region and their proportions are summarized in Table 1.

^1^H NMR spectrum of the reaction mixture (Figure 4) showed the presence of a major dimer product (~50%) corresponding to the quasi doublets (4.76 ppm and 4.48 ppm, Dimer **1**) that was very similar to *syn* HH dimers observed in cinnamic acid (CA) and other arylacrylic acid dimers that we have previously explored for CMP [18,20]. Another signal of dimeric product of lower, yet significant, proportion was observed downfield (4.97 ppm and 4.85 ppm, Dimer **2**), which was very similar in multiplicity pattern and chemical shift in *anti* HT dimers of CAs and AAs. The third, minor, dimer (~14%, Dimer **3**) with signals upfield was similar in quasi doublet pattern. The relative signal intensity of dimeric products and monomers (*cis* and *trans*) showed that reaction conversion of higher than 90% was attained, with the reaction directed predominantly towards the dimerization pathway with negligible isomerization. The dimers from the reaction mixtures were isolated, purified, and characterized spectroscopically (Supplementary Materials Section S3).

***^1^H NMR analysis of dimer signals:*** The three dimers present in the reaction mixture from CMP of **1a** in γ-CD were identified based on the chemical shifts and coupling constants reported by Montaudo et al. [28], and structures established in our own pursuits [18,20]. Table 2 presents the comparative analysis of the signals of dimeric products resulting from CMP and that of literature-reported values of dimers produced through solid-state, molten-state, other restricted molecular environments. Based on the direct comparison, the identities of Dimer **1** and Dimer **2** were in fact the *syn* HH and *anti* HT dimers as predicted; the identity of Dimer **3** was determined to be *anti* HH.

**Figure 5:**
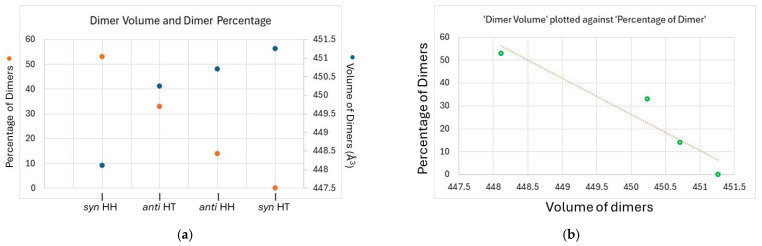
(**a**) Double axis plot of volume of dimers vs. dimer stereochemistry vs. percentage of dimers, and (**b**) percentage of dimer vs. volume of dimers showing an inverse relationship.

**Figure 6:**
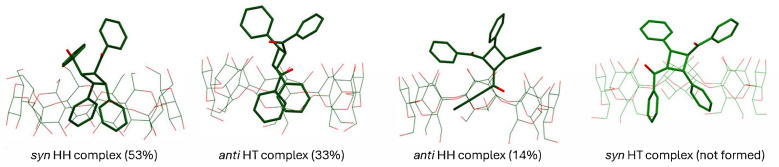
Energy-optimized structures of the four dimers of chalcone complexed to γ-CD performed in gas phase at HF 3-21G* in Spartan ’20, Version 1.1.4. Atoms are color-coded for elements: red atoms are oxygen, blue atoms are nitrogen, and dark green atoms are carbon.

***Computational chemistry:*** was used to analyze the structural features of the dimers to rationalize the selectivity observed. Given the limited cavity volume of the host, we hypothesized that product formation was influenced by the overall size of the dimer. The volume of the dimers was calculated in Spartan ’20, Version 1.1.4 in gas phase HF 3-21g* and compared to the percentage of dimers observed in the reaction mixture, which are plotted against each other (Figure 5). A clear correlation between the volume of dimer and percentage of observed product emerged, which validated our hypothesis that the smaller and more compact dimer is favored. The correlation is apparent from the dual axis plot of volume vs. dimer stereochemistry vs. percentage where an approximately linear trend between the variables was observed with opposite slopes; similarly, a volume vs. product distribution plot yielded a straight line with negative slope indicating an inverse relationship between dimer volume and its predominance (selectivity). Computed structures of the dimers included within the γ-CD cavity also provided a visual confirmation of this inference, as the two smallest dimers—the *syn* HH and *anti* HT dimers—were deeply embedded within the cavity due to their compactness, while *anti* HH and *syn* HT dimers were only partly included (Figure 6).

The computational chemistry analysis of the structures of the reactants’ ternary complexes (chalcone_2_@γ-CD) were also performed at higher level of theory. Energy optimized structures of the 2:1 complexes of chalcone in all four pre-reaction orientations that would yield the corresponding dimers were calculated at B3LYP D3/6-31G* in the gas phase (Supplementary Materials). The dipole moments of the reactant complexes resulting from the optimized structures (Table 3) provided key insights regarding the product selectivity observed in the reaction. Among the four pre-reactive isomeric complexes leading to the corresponding dimers, the order of dipole moment in the decreasing orders was *syn* HH > *anti* HH > *syn* HT > *anti* HT. Interestingly, the *syn* HH reactive orientation possessed the highest dipole moment, with significantly higher magnitude to that of the next reactive arrangement in the order. The coincidence of the *syn* HH predominating in the product distribution at room temperature with its highest dipole moment in both gas and aqueous phase suggests a causal relationship, as polar aqueous medium used in the complexation would favor the formation of a high dipole assembly.

Job’s method of continuous variation was performed using UV-Vis spectroscopy by mixing γ-cyclodextrin and chalcone 1a in varying mole fractions while maintaining constant total concentration (1.0 × 10^−4^ M, Supplementary Materials Figure S5). The Job’s plot was constructed by plotting the product of the mole fraction of γ-CD and the corresponding change in absorbance (ΔA·χ) at λmax versus the mole fraction yielded a plot with maxima at 0.5 indicating a binary (1:1) complex as the predominant species in dilute solution. This stoichiometry differs from that inferred from the photochemical experiments of 1a and 1b, where the reactive assembly is proposed to involve a ternary (2:1, guest:host) complex. This difference is attributed to the markedly different experimental conditions. The Job’s plot reflects dilute, thermodynamic equilibrium in homogeneous solutions, whereas the photochemical reactions are conducted at significantly higher effective concentrations under slurry conditions. Under such conditions, enthalpically stabilized higher-order assemblies can become accessible, even if they are not the dominant species in dilute equilibrium. Thus, the apparent difference in stoichiometry reflects concentration-dependent supramolecular organization rather than a discrepancy between methods.

***Analysis of product distribution***: presented in Table 1 based on the deduced structure of isomeric dimers, computational analysis of the complexes, and complex titration confers insights about the supramolecular factors directing product selectivity. The formation of *syn* HH as major product within γ-CD as well as CB8 coupled with the computational volume analysis suggests that the compactness of the dimer is perhaps the primary factor governing selectivity; the *syn* HH dimer is also the near exclusive dimer observed in CAs [18,19,29] and coumarins [30,31,32]. While CAs, coumarins and other aryl acrylic acids with strongly polar groups yielded only one dimeric product, chalcones yielded three dimers. This difference could be attributed to the stabilizing interactions such as hydrogen bonding and p-p interactions in HH arrangement and the lack of any such interactions in the HT arrangement. In chalcones, however, there are no such strongly stabilizing interactions in the HH stacking, and p-p interaction is potentially possible in both HH and HT arrangements, which explains the difference. Another interesting observation that stands out is the very strong favorability for *syn* HH dimer within CB8, about 23% more than that in γ-CD (Supplementary Materials Section S4). This difference could be attributed to the longer longitudinal dimension of CB8 (9.1 Å) compared to γ-CD (7.8 Å), and a narrower portal dimension (6.9 Å to 9.5 Å). The narrower CB8 portal favors a tighter stacking arrangement while the longer cavity allows for host’s extended structural enforcement on the chalcones resulting in a remarkably higher product selectivity in favor of *syn* HH. The reaction of chalcones within *b*-CD yielded exclusively isomerization product suggesting a 1:1 complex formation, as well as lack of reaction outside the cavity for both smaller (β-CD) and larger (CB8, γ-CD) cavitands. Chalcones were also photochemically unreactive in the solid state, likely due to topochemical constraints or insufficient molecular mobility within the crystal lattice to permit the structural rearrangement required for [2+2] PCA.

**Figure 7:**
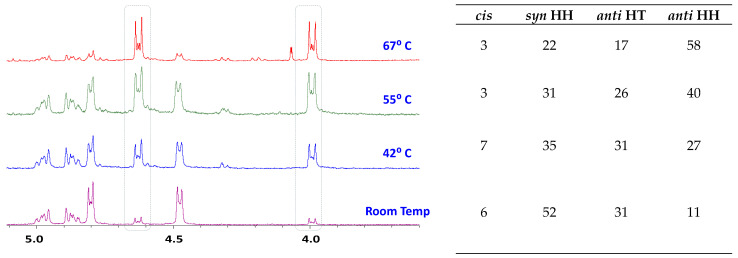
^1^H NMR spectra of reaction mixture showing shift in product selectivity resulting from photoexcitation of parent chalcone complexed to γ-CD (1a_2_@γ-CD) at different temperatures. The increase in *anti* HH (dashed boxes) dimer signals with increase in temperature represents a temperature-dependent switch in product stereoselectivity.

***Temperature-Dependent CMP:*** Due to the varying proportion of multiple dimers formed, we speculated that temperature could potentially, and significantly, influence the product distribution. The ternary complex slurry of **1a**_2_@γ-CD was prepared and split into four equal parts and irradiated simultaneously, each of the complex parts were irradiated while placed on a stirring hot-plate maintained at four different temperatures (room temp ~21 °C, 42 °C, 55 °C, and 67 °C, Figure 7). As expected, there was a noticeable change in product selectivity of the reaction observed in the ^1^H NMR of the reaction mixtures. However, to our surprise the product selectivity did not shift towards an even distribution; instead, the *anti* HH, the dimer that was formed in lowest amount among the three, was seen to increase with increasing temperature. The *anti* HH dimer proportion nearly sextupled, which is a remarkable and interesting change.

To rationalize this observed molecular behavior, we examined the dipole moments of the pre-reactive ternary (2:1 guest:host) complexes calculated at the B3LYP D3/6-31G* level in both gas phase and aqueous continuum (Table 3). Among the four possible arrangements, the *syn* HH complex exhibited the largest dipole moment, followed by the *anti* HH complex. While dipole magnitude alone may not dictate product distribution, it provides a measure of overall polarity and potential interaction with the aqueous environment. Importantly, structural analysis of the optimized complexes reveals that the *anti* HH arrangement is not fully encapsulated within the γ-CD cavity; instead, one aromatic moiety extends closer to the portal region, allowing partial exposure to the surrounding medium.

At lower temperatures, tighter encapsulation and enthalpic stabilization of compact assemblies such as *syn* HH likely dominate complex distribution. As temperature increases, entropic contributions and enhanced host movement become more significant. Thus, partially exposed arrangements such as *anti* HH, as seen in Figure 6, may be favored due to reduced confinement penalties. This, in combination with the comparatively high polarity of this less-confined structure, may favor the *anti* HH arrangement at elevated temperature. Thus, the temperature-dependent increase in *anti* HH formation may reflect a subtle balance between steric compactness, dipole-driven solvation effects, and dynamic reorganization of the ternary assembly. While this interpretation is consistent with the computational and structural data, the precise mechanistic origin of the temperature effect remains an open question that we intend to pursue in a more-focused study.

The observed temperature-dependent shift in selectivity likely reflects changes in the equilibrium population and geometry of the 2:1 host–guest complex. Similar temperature effects have been documented in γ-CD-mediated photocycloaddition of 2-anthracenecarboxylic acid [23], where product ratios were strongly governed by sterically defined stacking arrangements of rigid anthracene chromophore. In our work, the chalcone system examined herein involves a more flexible and electronically less differentiated substrate and increasing temperature shifts selectivity toward the larger anti-HH dimer, consistent with its comparatively higher dipole moment and partial exposure within the host cavity.

**Figure 8:**
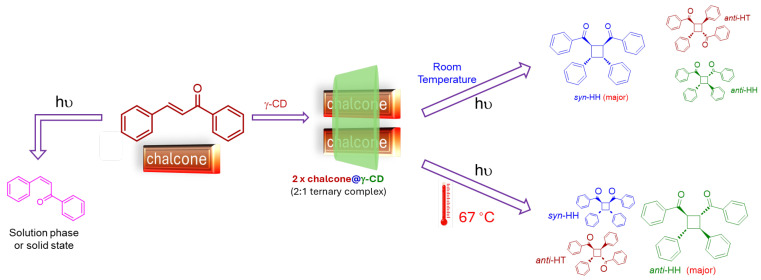
Summary of selectivity of chalcone photochemistry affected by supramolecular and temperature.

Based on the experiments performed, and the reaction outcomes, an interesting picture about the CMP of chalcones emerge, especially when compared to the CMP of cinnamic acids, alkyl cinnamates, aryl acrylic acids, and coumarins. While all the previously explored photoactive guests yielded a single dimer as the major product, chalcones yielded a mixture of products. This is due to the strong disparity in the molecular features of the guest between the head and tail ends, which dictates their inter-molecular orientation. Whereas in case of chalcones, the head and tail-ends of the guest are of similar polarity, which leads to a lack of significant difference between the different precursory complex structures, which manifests as the formation of multiple dimers. Moreover, this distribution, due to their close energetic similarity, is easily affected by the change in temperature, which affords the means to switch selectivity from one dimer to the other. The behavior of the system is summarized in Figure 8.

## 4. Experimental Section

***General Procedures:*** All chemicals and solvents, including chalcones and γ-CD, were purchased from commercial suppliers (MilliporeSigma, St. Louis, MO, USA and TCI America, Portland, OR, USA), and used as received without further purification. Solvents were of analytical grade and used as received. Host–guest complexes were prepared by sonicating a mixture of chalcone (typically 50 mg) and γ-cyclodextrin (0.5 equivalents) in 5 mL distilled water for two hours at approximately 50 °C. The suspension was then stirred for an additional four hours at room temperature. A stable white slurry, indicative of inclusion complex formation, was obtained and used directly for irradiation experiments.

***Irradiation Procedure:*** Water was removed from the aqueous slurry of the chalcone_2_@γ-CD inclusion complex by filtering it through a fritted Buchner funnel and the powder dried under vacuum for two hours and then air dried for 24 h. About 0.1 g of the dried complex was then transferred onto a Pyrex glass plate to form a thin layer sandwiched between Pyrex glass plates. The slurry was then irradiated using a medium-pressure mercury vapor lamp (450 W) housed in a water-cooled Pyrex immersion jacket to filter short-wave UV light. Irradiation was conducted for a period of 24 h under continuous stirring. Temperature-dependent experiments were performed by placing the glass plates on a hotplate stabilized at required temperatures; the temperature of the hotplate was determined by measuring temperature of a beaker water bath placed alongside the sample on the hotplate. Photoreaction progress was visually monitored by observing the discoloration of the slurry, indicative of loss of conjugation. The conversions were further confirmed using ^1^H NMR spectroscopy.

***Extraction and Purification:*** Following irradiation, the solid complex was dispersed in water and extracted multiple times with ethyl acetate. The combined organic extracts were dried over anhydrous Na_2_SO_4_ and concentrated under reduced pressure to yield the crude photoproduct mixture. Further purification of dimers was accomplished by flash chromatography using silica gel (60–230 mesh) with ethyl acetate/hexane mixtures as eluents.

***NMR Analysis:*** The photoproduct mixtures were analyzed by ^1^H NMR spectroscopy on a Bruker Avance 300 MHz spectrometer (Billerica, MA, USA) in CDCl_3_. Chemical shifts are reported relative to referencing CDCl_3_ solvent signal at 7.27 ppm. Identification and quantification of dimeric products was performed based on distinctive cyclobutane proton resonances and coupling constants, and compared against values reported in the literature.

***Computational Studies:*** Computational analyses of chalcone dimers included in γ-CD cavities were carried out using Spartan ‘20 software, Version 1.1.4. Geometry optimizations were initially performed using Hartree–Fock methods with the 3-21G* basis set in the gas phase to obtain structural parameters and volumetric data. Subsequently, the pre-reactive ternary (2:1 guest:host) complexes were further optimized at the B3LYP D3/6-31G* level to compute dipole moments and assess electronic characteristics. Relative stabilities of the complexes were assessed based on computed energies, and molecular volumes were used to correlate spatial constraints with observed experimental selectivities.

***Temperature-Dependent Studies:*** To investigate temperature effects, inclusion complexes were similarly prepared and divided into equal portions. Each portion was irradiated under identical conditions but at different controlled temperatures (21 °C, 42 °C, 55 °C, and 67 °C). Product distributions were analyzed using ^1^H NMR as described previously, providing insights into temperature-induced variations in stereochemical selectivity. Detailed experimental procedure is provided in supporting information document (Supplementary Materials Section S1).

## 5. Conclusions

In this work we report our study on the cavitand-mediated photodimerization of chalcones within γ-CD cavities, exploring the supramolecular, photochemical, and external influences on the selectivity of the reaction. We have demonstrated that the CMP approach significantly enhances the efficiency and selectivity of the [2+2] photodimerization process compared to the reaction in homogeneous media and organized media like the solid state. Unlike cinnamic acids and other aryl acrylic substrates that were studied using the CMP method, chalcones formed multiple dimers due to their inherent structural symmetry and flexibility leading to diverse spatial orientations within the host cavity. Computational modeling indicates that the dimer formation was strongly influenced by spatial constraints, favoring smaller, more compact dimers such as the *syn* HH and *anti* HT configurations. Temperature-dependent experiments revealed a pronounced shift towards the *anti* HH dimer at elevated temperatures; together with the dipole moment calculations of the pre-reactive ternary assemblies, this highlights the role of polarity and subtle supramolecular effects in governing product distribution. This atypical temperature responsiveness presents opportunities for controlled stereochemical tuning in photochemical syntheses. Overall, the findings underscore the versatility of macrocyclic hosts in directing photochemical reactions, while also advancing fundamental understandings of supramolecular influences on chemical reactivity.

## Figures and Tables

**Table 1:** Product distribution of chalcones irradiated in various media and constrained molecular environments.

Reactant	Medium/Constraint	Product %				%Conversion
		*cis*	*syn* HH	*anti* HT	*anti* HH	
**1a**	Methanol	100	-	-	-	61
**1a**	β-CD	100	-	-	-	59
**1a**	γ-CD	6	52	31	11	86
**1a**	CB8	6	84	4	6	78
**1b**	Methanol	100	-	-	-	73
**1b**	β-CD	100	-	-	-	73
**1b**	γ-CD	5	60	28	7	82

Product percentages and conversions are reported as ±5% from three independent experiments. Photoirradiation of **1a** and **1b** in solid state showed no noticeable product formation or conversion. Duration of irradiation for which the conversion is reported is 24 h.

**Table 2:** Compilation of ^1^H NMR data of dimer: chemical shifts and coupling constants.

Dimer Stereochemistry	Cyclobutane ppm		Coupling Constant	
	Literature	This Work	Literature	This Work
*syn* HH		(H_2_) 4.76, (H_1_) 4.48		*J*_H1–H2_ 6.25 Hz
*anti* HT		(H_3_) 4.97, (H_4_) 4.85		*J*_H3–H4_ 17.67 Hz
*anti* HH	4.67, 3.97	(H_6_) 4.63, (H_5_) 3.99	8.8 Hz	*J*_H5–H6_ 8.85 Hz
*syn* HT		Not formed		*J*_H7–H8_ N/A
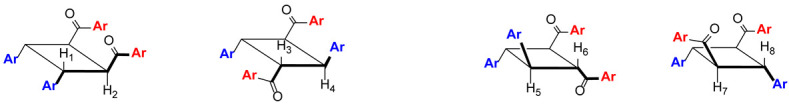				

**Table 3:** Dipole moments of the pre-reactive arrangements of ternary (chalcone_2_:γ-CD) complexes calculated in B3LYP D3/6-31G* in gas phase and aqueous medium.

Medium	*syn* HH	*anti* HH	*syn* HT	*anti* HT
Gas phase	7.9298	5.1081	3.5912	2.5816
Aqueous	11.9313	6.5641	6.0108	3.5872

## Data Availability

The original contributions presented in this study are included in the article and Supplementary Materials. Further inquiries can be directed to the corresponding author.

## Supplementary Materials

The following supporting information can be downloaded at: https://www.mdpi.com/article/10.3390/molecules31060983/s1, Figure S1. Spectral representation of complexation of chalcone 1a in D_2_O and the same in presence of 0.5 equivalents of with γ-cyclodextrin. Figure S2. NMR spectra (^1^H and ^13^C) of purified dimers obtained from the photodimerization reactions in CDCl_3_. Figure S3. ^1^H NMR of reaction mixture of chalcone 1a complexed to CB8 (1a_2_@CB8) irradiated (room temp) in as a slurry showing formation of higher proportion of *syn* HH compared to that observed in γ-CD in CDCl^3^. Figure S4. Stacked spectra of reaction mixture resulting from CMP of chalcone in γ-CD and CB8 showing stark difference in product selectivity in favor of *syn* HH in CB8. Figure S5. UV–Vis absorption spectra of chalcone in the presence of increasing mole fractions of γ-cyclodextrin at a constant total concentration of 1.0 × 10^−4^ M. Figure S6. Job’s plot for the chalcone-γ-cyclodextrin complex formation from the UV-Vis titration presented in Figure S5. Table S1. Volume of Photodimers Obtained from Calculations performed in HF 3-21G*.
