# Supplementary material for: Cavitand-Mediated Photodimerization of Chalcones: The Effect of Supramolecular Influences and Temperature on Reaction Selectivity
Source: Molecules. 2026 Mar 15;31(6):983. doi: 10.3390/molecules31060983 (PMC13028674; doi:10.3390/molecules31060983)
Supplement: Supplementary file 1 [file molecules-31-00983-s001.zip › molecules-4145277-supplementary.pdf]

Cavitand-Mediated Photodimerization of Chalcones: The Effect of Supramolecular Influences and Temperature on Reaction Selectivity

Joydip Chatterjee [1], Mahesh Pattabiraman [1,*], Debajit Chakraborty [2], Aleksander L. Wysocki [2] and Frank Kovacs [1]

[1] Department of Chemistry, University of Nebraska at Kearney, Kearney, NE 68849, USA
[2] Department of Physics and Astronomy, University of Nebraska at Kearney, Kearney, NE 68849, USA
* Correspondence: pattabiramm2@unk.edu

Page No.	Section No.	Description
2	Section S1	Experimental Procedures
3	Section S2	Complexation titration, ^{1}H NMR
3 to 5	Section S3	NMR spectra of purified compounds
6	Section S4	NMR spectra of mixture of irradiation of **1a**$_2$@γ-CD
7	Section S5	Job's Plot
8	Section S6	Table of dimer volumes

Section S1: Experimental Procedures

Complexation Procedure Approximately 10 mg of chalcone (1a or 1b) was weighed into a 20 mL scintillation vial, and 0.5 equivalents of γ-cyclodextrin (γ-CD) were added. To this, 5 mL of distilled water and a small magnetic stirring bar were introduced. The mixture was heated to approximately 50 °C on a stirring hotplate and sonicated for 2 hours to aid dissolution of the sparingly soluble chalcone. After sonication, the mixture was stirred at 50 °C for an additional 3 hours and then allowed to stir at room temperature for another 4–5 hours. The initially heterogeneous mixture gradually formed a uniform, chalky white slurry, indicative of ternary inclusion complex formation ($chalcone_2$@γ-CD). This complex was used directly in photochemical experiments without further purification.

Irradiation Procedure The aqueous slurry of the inclusion complex was transferred to a Pyrex scintillation vial and irradiated using a medium-pressure mercury vapor lamp (450 W) in a water-cooled Pyrex immersion jacket. Irradiation was typically carried out for 24 hours with continuous stirring. The progress of the photoreaction was monitored by visual decoloration of the slurry, indicating the disruption of the conjugated chromophore system.

Decomplexation and Extraction After irradiation, the reaction mixture was transferred to a 125 mL Erlenmeyer flask and diluted with 50 mL of water. To this, 30 mL of ethyl acetate was added, and the mixture was vigorously stirred with a magnetic stir bar for 2 hours. This process disrupts the inclusion complex and facilitates transfer of the organic products into the ethyl acetate phase. The organic layer was separated, dried over anhydrous sodium sulfate, and concentrated under reduced pressure using a rotary evaporator. The residue was further dried under vacuum and used for spectroscopic analysis.

Chromatographic Separation The crude photoproduct mixture was dissolved in dichloromethane and analyzed by thin-layer chromatography (TLC) using silica gel plates. Elution was performed with varying concentrations of dichloromethane/hexane (typically 15–50%) depending on the polarity of the starting chalcone. Resolved spots were isolated by cutting the TLC plate and scraping off the silica. Each band was eluted with methanol and filtered using a fritted funnel to remove silica. The methanol solutions were rotary evaporated and dried under vacuum. Residual TLC binding agents sometimes appeared in NMR as a singlet near 5.3 ppm.

NMR Spectroscopy ^{1}H NMR spectra of photoproducts were acquired using a Bruker Avance 300 MHz spectrometer at room temperature in $CDCl_3$. Dimer structures were assigned based on chemical shifts and coupling constants of the mid-field cyclobutane proton signals and were compared to literature-reported values and prior CMP studies. Complexation titration studies were performed in D_2O.

NMR Titration of Chalcone with γ-CD To evaluate the inclusion complexation, 5 mg of chalcone 1a was dissolved in 0.5 mL of D_2O by titration with small aliquots (~10 µL) of NaOD until fully dissolved, generating the 1a–Na^+ salt. A stock solution of γ-CD in D_2O was then added incrementally, and ^1H NMR spectra were recorded before and after each addition to monitor upfield shifts and signal broadening indicative of complex formation.

Computational Analysis Computational modeling of chalcone dimers and ternary host–guest complexes was performed using Spartan '20. Geometry optimizations of isolated dimers were carried out at the HF/321G* level in the gas phase to obtain volumetric data for correlation with experimental product distributions. For the pre-reactive ternary (2:1 guest:host) complexes, geometry optimizations were subsequently performed at the B3LYP-D3/6-31G* level to obtain dipole moments and relative electronic characteristics. These calculations were conducted in both gas phase and aqueous continuum where indicated.

Temperature-Dependent Photodimerization To evaluate temperature effects on stereochemical selectivity, identical inclusion complexes of $1a_2$@γ-CD were split into four vials and irradiated under identical conditions at different temperatures (21 °C, 42 °C, 55 °C, and 67 °C). Reaction mixtures were extracted and analyzed by ^{1}H NMR as described above, and relative dimer ratios were compared across temperatures.

Section S2: Inclusion Complex Titration

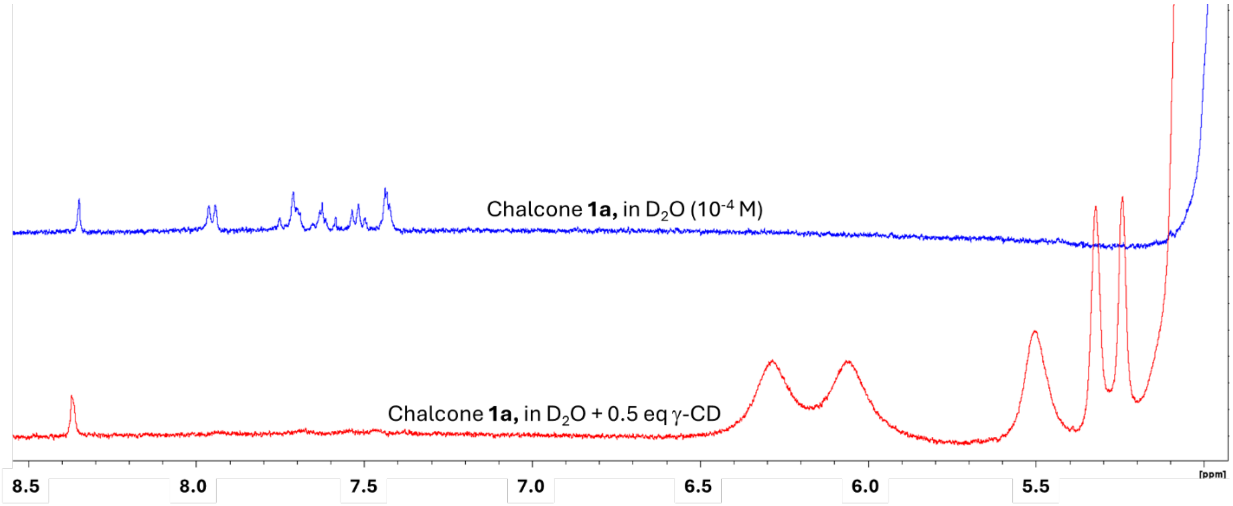

Figure S1: Spectral representation of complexation of chalcone **1a** in D_2O and the same in presence of 0.5 equivalents of with γ-cyclodextrin.

Section S3: NMR spectra of purified compounds

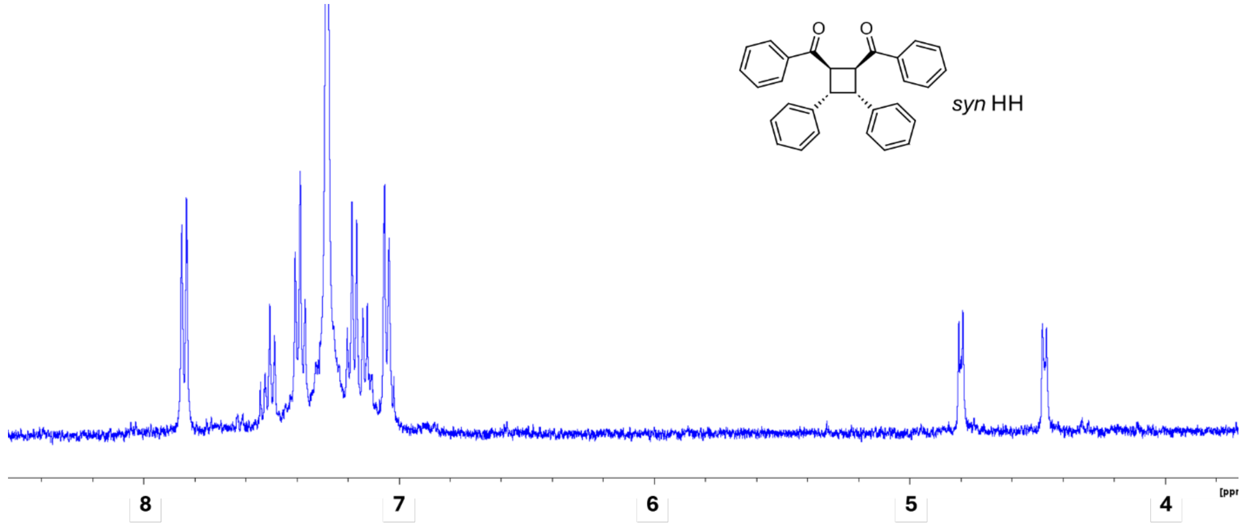

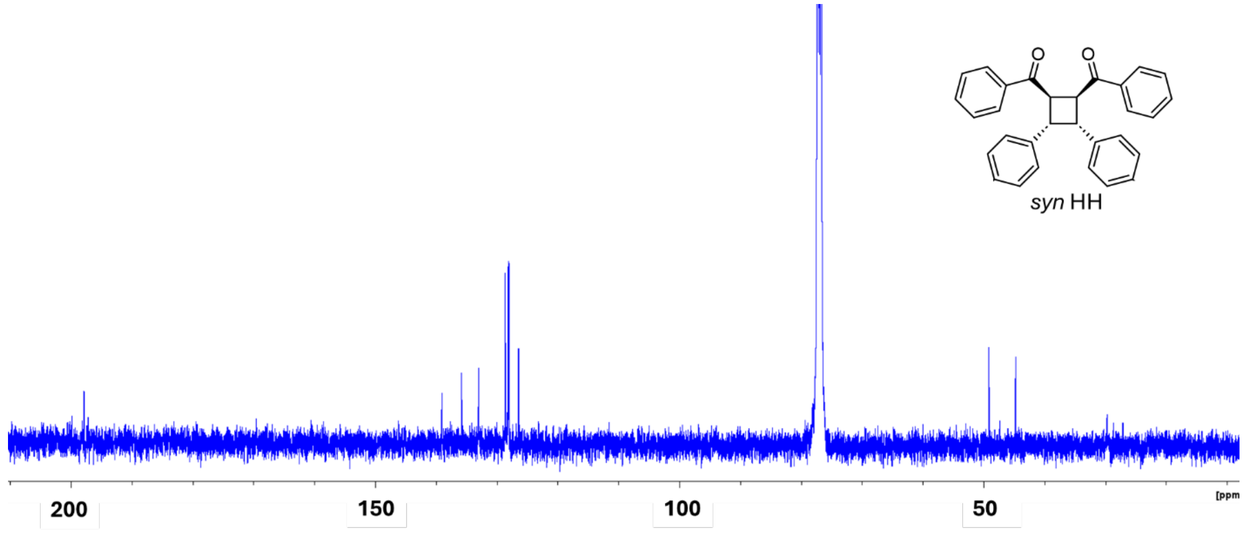

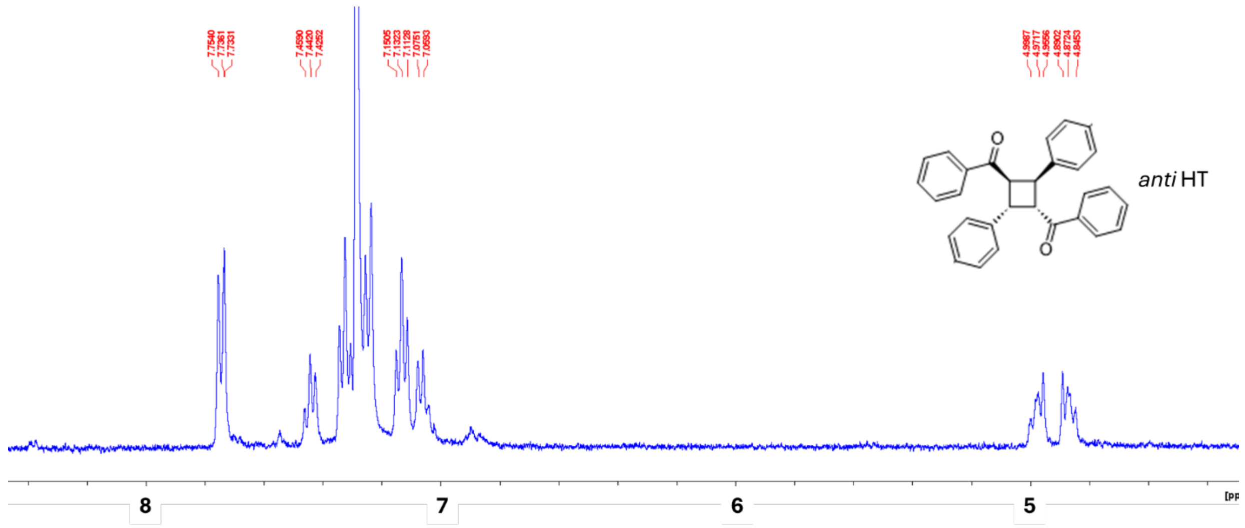

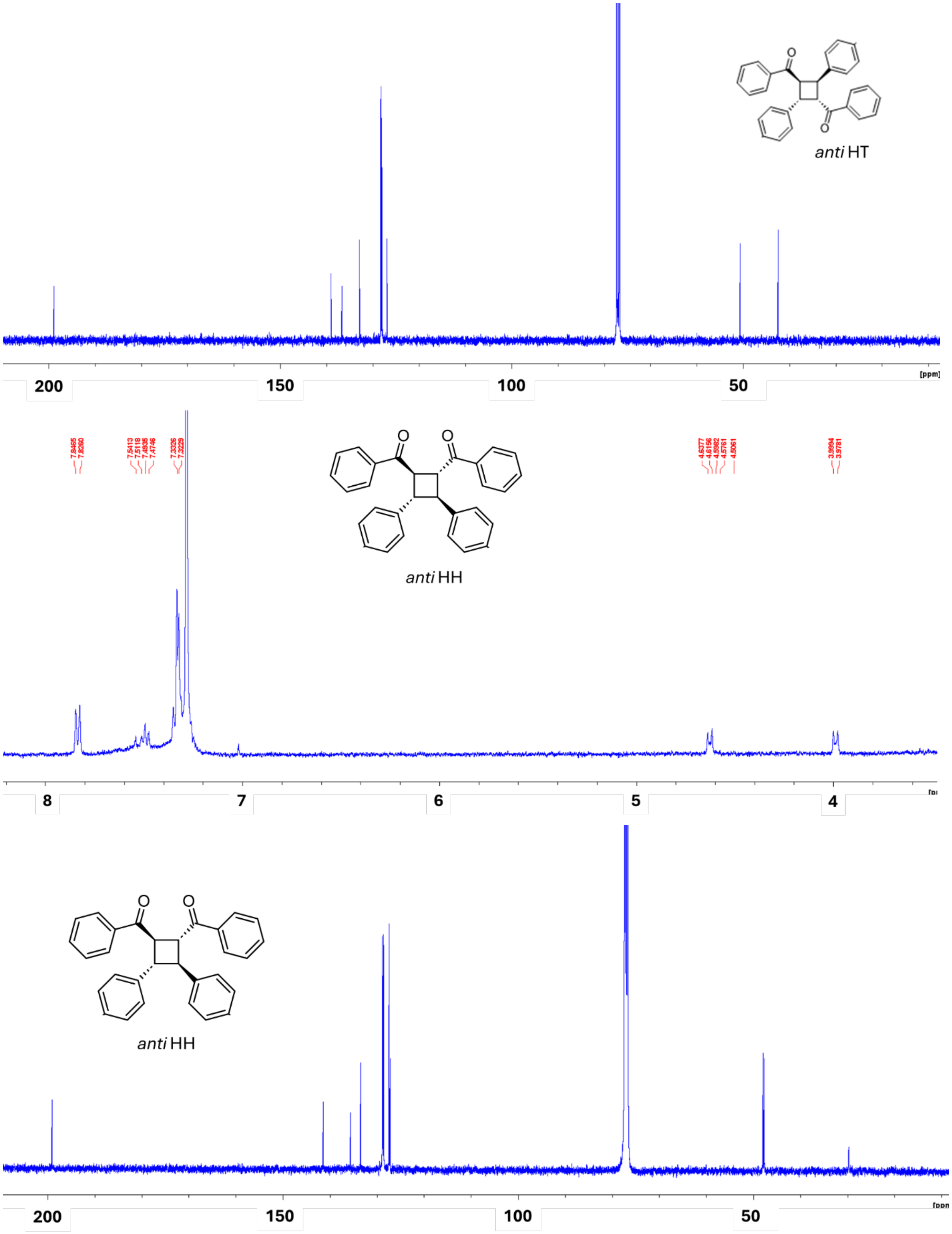

Figure S2: NMR spectra (^{1}H and ^{13}C) of purified dimers obtained from the photodimerization reactions in $CDCl_3$.

Section S4: CB8 complex reaction mixture

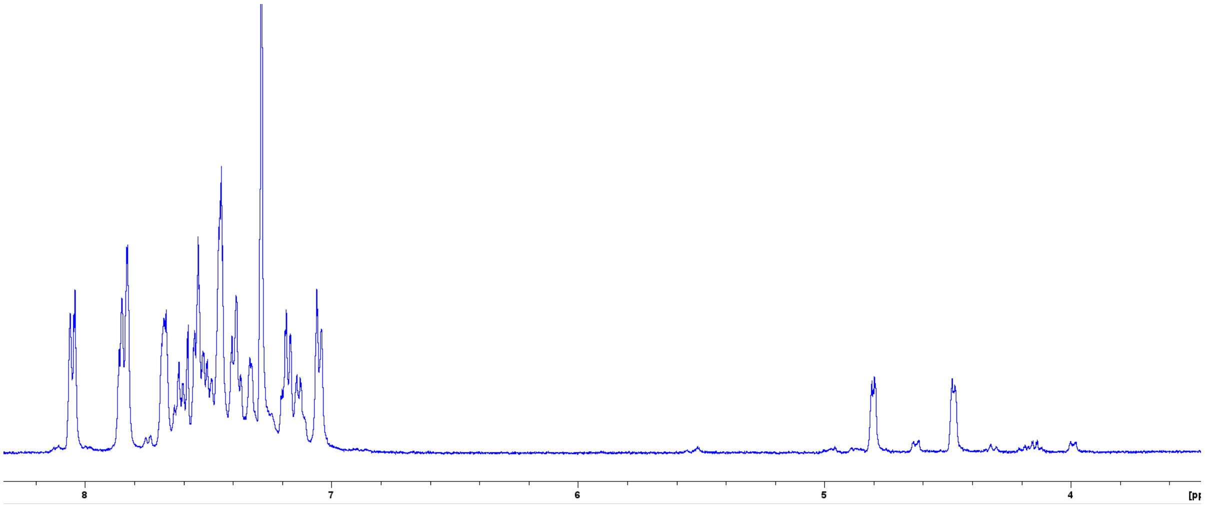

Figure S3: ^{1}H NMR of reaction mixture of chalcone **1a** complexed to CB8 (**1a**$_2$@CB8) irradiated (room temp) in as a slurry showing formation of higher proportion of *syn* HH compared to that observed in γ-CD in $CDCl^3$.

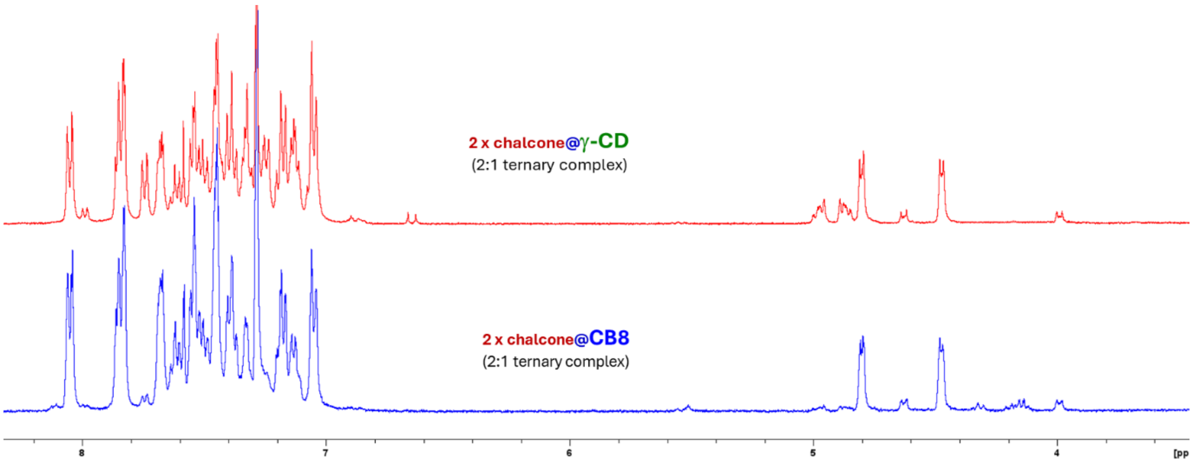

Figure S4: Stacked spectra of reaction mixture resulting from CMP of chalcone in g-CD and CB8 showing stark difference in product selectivity in favor of *syn* HH in CB8.

Section S5: Job's Plot

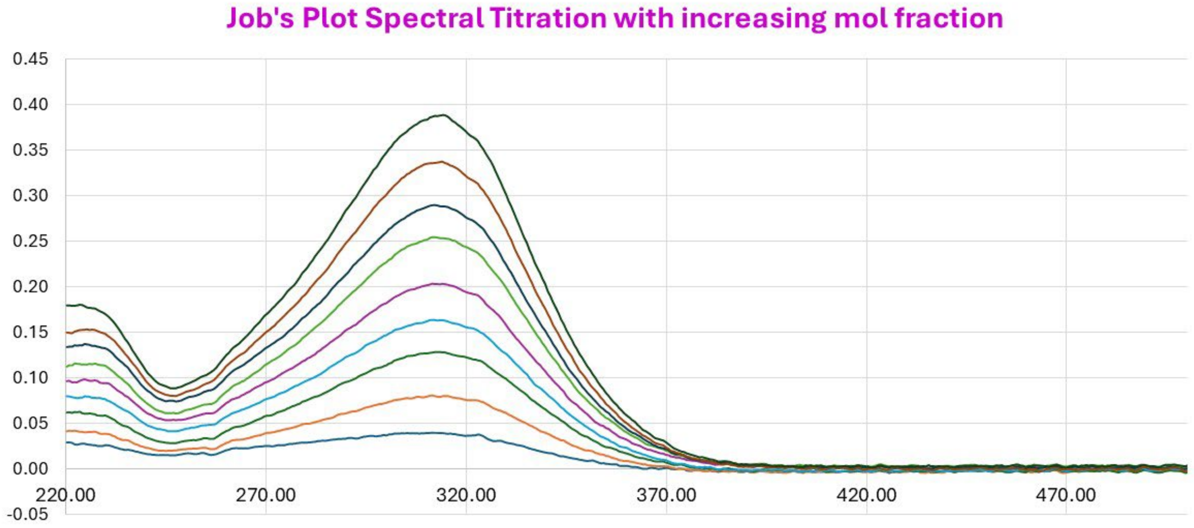

Figure S5: UV–Vis absorption spectra of chalcone in the presence of increasing mole fractions of γ-cyclodextrin at a constant total concentration of 1.0 x 10^{-4} M.

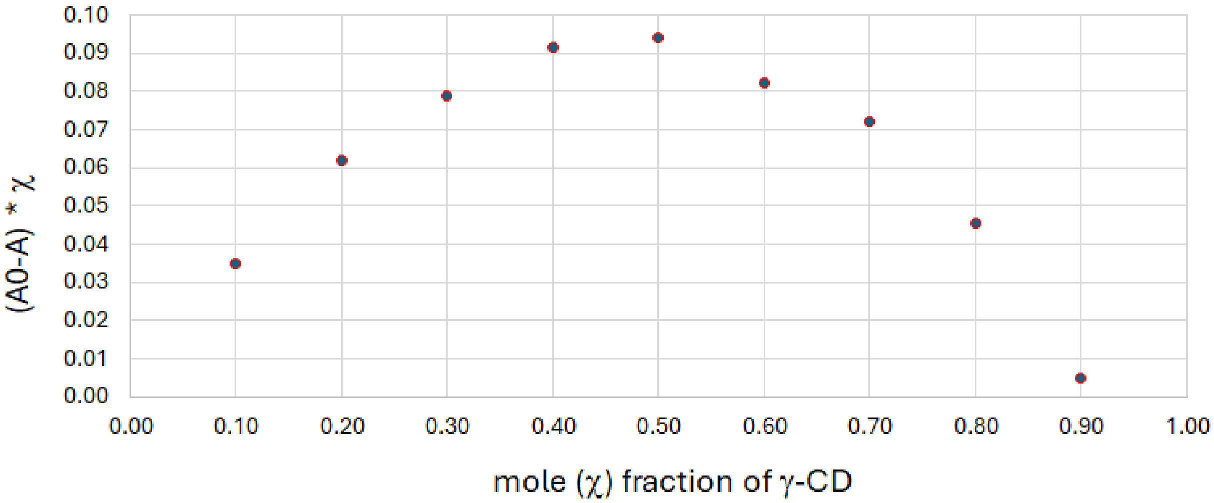

Figure S6: Job's plot for the chalcone-γ-cyclodextrin complex formation from the UV-Vis titration presented in SI Figure 5. The graph plots continuous variation function vs mole fraction of chalcone using absorbance changes measured at λ_{max} (310 nm).

Section S6:

Table S1 Volume of Photodimers Obtained from Calculations performed in HF 3-21G*

**Dimer**	*syn* HH	*anti* HT	*anti* HH	*syn* HT
**Volume (Å^3)**	448.11	450.24	450.71	451.26
